# A new tool to support the protection of workers’ health: development and application of an innovative analytical method for biomonitoring occupational exposure to formaldehyde

**DOI:** 10.1007/s00216-026-06461-x

**Published:** 2026-03-31

**Authors:** Arianna Antonucci, Matteo Albano, Ivano Pindinello, Leila Fabiani, Claudia Cipollone, Giada Mastrangeli, Riccardo Mastrantonio, Mario Muselli, Cinzia Lucia Ursini, Delia Cavallo, Marta Petyx, Giorgia Di Gennaro, Giuseppe De Palma, Matteo Vitali, Carmela Protano

**Affiliations:** 1 Department of Public Health and Infectious Diseases, Sapienza University of Rome, P.le Aldo Moro 5, 00185 Rome, Italy; 2 Department of Life, Health and Environmental Sciences, University of L’Aquila, P.le Salvatore Tommasi 1, Coppito, 67100 L’Aquila, Italy; 3 Department of Occupational and Environmental Medicine, Epidemiology and Hygiene, Italian Workers’Compensation Authority-INAIL, Monte Porzio Catone, 00078 Rome, Italy; 4 Unit of Occupational Health and Industrial Hygiene, Department of Medical and Surgical Specialties, Radiological Sciences and Public Health, University of Brescia, Piazzale Spedali Civili 1, 25123 Brescia, Italy

**Keywords:** Human biomonitoring, Occupational exposure, Gas chromatography–tandem mass spectrometry, Headspace solid-phase microextraction, Formaldehyde, Urine

## Abstract

Formaldehyde (FA) is a widely used industrial chemical and a ubiquitous environmental contaminant, for which biological monitoring remains challenging due to its high reactivity and substantial endogenous background. In the present study, a sensitive and selective analytical method based on headspace solid-phase microextraction with on-fiber derivatization (HS-SPME) coupled to gas chromatography–tandem mass spectrometry (GC–MS/MS) was developed and applied for the determination of urinary FA. Direct on-fiber derivatization using O-(2,3,4,5,6-pentafluorobenzyl) hydroxylamine (PFBHA) was optimized to ensure quantitative derivatization, reproducibility, and minimal sample handling. The method was validated in terms of linearity, sensitivity, precision, and robustness, demonstrating suitability for trace-level FA determination in complex biological matrices. The applicability of the method was evaluated in an occupational biomonitoring campaign involving 124 subjects, including workers occupationally exposed to FA (healthcare personnel, laboratory staff, and firefighters) and non-exposed administrative employees. Urinary FA concentrations showed wide inter-individual variability and substantial overlap between exposed and non-exposed groups, particularly at low exposure levels. Stratified analyses indicated associations with sex and smoking habits, while age and body mass index showed no clear influence. Overall, the results highlight both the analytical reliability of the proposed HS-SPME GC–MS/MS approach and the biological complexity of urinary FA as an exposure biomarker.

**Graphical Abstract:**

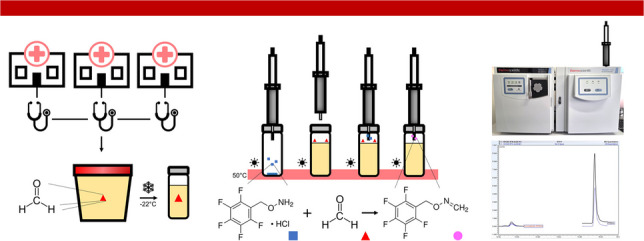

## Introduction

Formaldehyde (FA) is a simple, highly reactive aldehyde that plays a dual role as a ubiquitous environmental pollutant and as an industrial chemical. Indeed, FA is used in the manufacture of resins, adhesives, and disinfectants, and in the textile and wood-processing industries. Besides, it is also generated endogenously in humans through metabolic demethylation of various substrates [1]. The widespread use and presence of FA in the environment, together with its recognized adverse health effects [2], make it a chemical of major concern in occupational and environmental hygiene. Indeed, FA is a recognized sensory irritant compound, especially for sensitive individuals, and it is classified by the International Agency for Research on Cancer (IARC) as a Group 1 human carcinogen [3–5].

Occupational exposure to FA occurs via inhalation of vapor or aerosol in industrial settings, laboratories, medical-histology facilities, and other workplaces that handle FA-releasing substances [1]. Several methods have proven to be useful in mitigating occupational exposure to FA, including organization methods, engineering control methods, technical strategies, and the use of personal protective equipment, able to reduce airborne FA almost completely [6]. Nevertheless, these suitable procedures are not standardized and applied in all work settings, nor do they guarantee a complete elimination of FA from air; thus, it is essential to monitor occupational exposure to FA with appropriate procedures in order to assess and manage the related risk. Ambient and personal air sampling remain the standard methods for assessing exposure to FA in occupational settings [7]; however, these external measurements only estimate the internal dose and do not account for individual differences in absorption, metabolism, and excretion of the substance. Consequently, there is increasing interest in identifying reliable biomarkers of internal exposure, especially in biological matrices that are easy to collect and manage, such as urine, which may provide a more precise representation of absorbed and metabolized FA [8]. In particular, the development of a robust urinary biomonitoring method for evaluating occupational exposure offers several advantages, such as the possibility of using it as a non-invasive approach and as a potential tool for integration with routine health surveillance. Besides, it allows the use of a metric that reflects the biologically available internal dose rather than ambient concentration only and accounts for factors such as mitigation strategies, variable ventilation, use of personal protective equipment, dermal absorption, and metabolic differences. To achieve this goal, it is necessary to optimize and validate an analytical procedure that fulfils the criteria of sensitivity, selectivity, reproducibility, minimal sample preparation, rapidity, minimal use of chemicals, and reliable quantification in the urine matrix [9].

The quantification of FA or other suitable biomarkers for this specific substance in urine presents several analytical challenges. First, FA’s high reactivity and volatility mean it is rapidly converted to other species, including its major metabolite formate, or forms adducts with biomolecules such as glutathione, proteins, and amino acids [4, 10]. Besides, the presence of many potential interferents in urine matrix and the endogenous generation of FA and its metabolites, the dietary sources, and the inter-individual variation in metabolism can further complicate the interpretation of the results, as they may confound the determination and quantification of the specific biomarker. Despite these obstacles, a systematic review published in 2024 on biomarkers of FA occupational exposure found that only a few studies actually assessed biomarker levels of internal dose and emphasized the need for better analytical methods and validation in occupational settings [8]. Concurrently, regulatory bodies continue to update the toxicological assessment of FA: the United States Environmental Protection Agency (EPA) released the final IRIS Toxicological Review of Formaldehyde (Inhalation) in 2024, reinforcing the hazard profile of FA and underscoring the importance of exposure assessment in occupational environments [3].

Traditional analytical procedures for the determination of FA in biological matrices commonly involve derivatization followed by liquid–liquid extraction and chromatographic analysis. Although these methods can achieve acceptable sensitivity, they are often labor-intensive, require relatively large sample volumes and organic solvents, and may introduce additional sources of error during sample handling and transfer [11]. Several studies attempted to overcome these limitations by using methods based on solid-phase microextraction (SPME) coupled with gas chromatography–tandem mass spectrometry (GC–MS/MS) [12, 13]. SPME provides an attractive alternative by integrating sampling, extraction, concentration, and sample introduction into a single, solvent-free step. This technique minimizes matrix effects, reduces sample preparation time, and lowers the risk of analyte loss or contamination—factors that are particularly critical when dealing with volatile and reactive compounds such as FA. For instance, the use of HS-SPME with GC–MS/MS has been demonstrated for the quantification of low-level volatile organic compounds in urine, with detection limits in the sub-µg L^−1^ range, underscoring the feasibility of trace-level biomonitoring in biological matrices [14]. Coupling SPME with GC–MS/MS further enhances analytical performance by combining the high separation efficiency of gas chromatography with the exceptional selectivity and sensitivity of tandem mass spectrometry. The MS/MS configuration allows the specific detection of the FA derivative even in the presence of matrix interferences, ensuring accurate quantification at very low concentration levels. Moreover, recent biomonitoring reviews emphasize the analytical advantages of GC–MS/MS for internal dose assessment and highlight that urinary biomonitoring methods based on GC–MS/MS are increasingly being adopted for exposure assessment of environmental and occupational contaminants [15]. In addition, the combination of SPME and GC–MS/MS is particularly suitable for volatile or semi-volatile compounds following appropriate derivatization or trapping, which is relevant for FA due to its high reactivity and volatility [16].

In the present study, we report the optimization and validation of an analytical method for the determination of FA exposure through urine samples of workers. After validation in accordance with international guidelines (for example, accuracy, precision, limits of detection and quantification, recovery, stability), we applied the method to urine samples collected from both exposed and non-exposed workers in relevant occupational settings, thereby illustrating its practical applicability in risk assessment programs.

## Experimental

### Chemicals, reagents, and other materials

A formaldehyde solution in water 37% (10–15% methanol as stabilizer) and derivatization agent, O-(2,3,4,5,6-pentafluorobenzyl)-hydroxylamine hydrochloride (PFBHA) (99.0% powdered), were purchased from Sigma Aldrich (St. Louis, MO, USA). Analytical grade sodium chloride (NaCl) was supplied by Carlo Erba (Milan, Italy). Water used for standard dilutions was purified by a Smart NE Laboratory Ultrapure water system by Heal Force (Shanghai, China).

Polypropylene specimen containers for urine collection were supplied by VWR (Milan, Italy). Gastight syringes from Agilent (Santa Clara, CA, USA) and 2 mL amber glass vials with screw top from Supelco (Bellefonte, PA, USA) were used for standard solutions preparation.

20 mL glass vials, septa (silicone/PTFE, 20 mm diameter), and perforated aluminum seals, used for calibration standards and sample analyses, were purchased from Agilent (Santa Clara, CA, USA). SPME fiber holder for manual use and 65 μm polydimethylsiloxane/divinylbenzene (PDMS/DVB) fibers from Supelco (Bellefonte, PA, USA) were chosen for extracting PFBHA and for performing the subsequent on-fiber derivatization reaction/extraction of urinary FA.

### Analytical equipment

An aluminum block device (VWR, Milan, Italy) was used for providing agitation and temperature control during the incubation process and HS-SPME sampling-derivatization of FA from urine.

Analysis was performed by a Trace 1600 gas chromatograph coupled to a TSQ 9610 triple quadrupole mass spectrometer (Thermo Fisher Scientific, Rodano, Milan, Italy) running in electron impact ionization mode (EI, 70 eV, source at 320 °C). The mass spectrometer operated in MS/MS mode using high purity argon (5.0, purity grade ≥ 99.999% v/v) from Linde as collision gas at a pressure of 4.83 bar in the collision cell.

A PTV injector module equipped with a PTV siltek metal liner (0.75 mm diameter, Restek, Milan, Italy), was used for the desorption-injection operation of the investigated compounds. Chromatographic separation was performed on a HP-5MS capillary column (60 m × 0.25 mm × 2.5 μm film thickness; Agilent) employing helium (5.0, purity grade ≥ 99.999% v/v) from Linde as carrier gas. Chromeleon software (Thermo Fisher Scientific) was used for data acquisition/processing and instrument management.

### Standard preparation

The formaldehyde solution (37% by weight) was diluted in ultrapure water to obtain a standard solution with a concentration of 370 µg mL^−1^. This solution was freshly arranged and utilized for the preparation of calibration standards. A pool of urine collected from subjects not exposed to FA was utilized for the formulation of blanks and calibration standards. This urine matrix was utilized instead of purified water for compensating the matrix effect. Before use, the pool of urine samples was flushed with nitrogen gas (purity grade equal to 99.9999% v/v) from Alphagaz (Milan, Italy) and added with a portion of ultrapure water (urine:water = 2:1), with the objective of further reducing the potential concentration of the investigated analyte.

Blanks and calibration standards were prepared in a consistent manner as follows: 20 mL glass vials containing 3 g of dried NaCl were filled with 14 mL of urine matrix and sealed with Teflon-lined septa and holes aluminum caps; subsequently, the appropriate volumes of formaldehyde standard solution, ranging from 8 to 50 μL, were added to 14 mL aliquots of urine to obtain six calibration standards containing FA at concentrations of 0.21, 0.42, 0.63, 0.84, 1.06, and 1.27 µg mL^−1^. Each calibration level was prepared in triplicate and utilized to generate a calibration curve. Unspiked samples of the same pool of urine were kept as blanks. Three additional calibration standards at a concentration of 0.25, 0.53, and 0.95 µg mL^−1^ were prepared in a consistent manner to assess the repeatability and the accuracy of the method.

All blanks and calibration standards were prepared on the same day and stored at −20 °C until analysis, which was performed within 30 days of preparation. Prior to analysis, each blank, standard, or unknown urine sample was thawed at room temperature.

### Sampling “on-fiber derivatization” procedure

The analysis and quantification of FA in GC–MS require a derivatization step to make the reactive gas more stable and less polar, better suited for the GC process. A multitude of studies have demonstrated that O-(2,3,4,5,6-pentafluorobenzyl)-hydroxylamine hydrochloride (PFBHA) functions as an effective derivatizing agent in the analysis of FA through gas chromatography by increasing stability and improving detection [17–19]. The mechanism of this reaction involves the formation of the corresponding oxime, O-(2,3,4,5,6-pentafluorobenzil) formaldehyde oxime (FA-oxime), a product with great thermal and photostability. Several analytical methods using derivatization of aldehydes based on fiber SPME have proven useful in various fields of application [20–23]. In these studies, the alternative process of performing the derivatization, by adding PFBHA to the SPME fiber coating and then exposing this to the headspace of the sample, has been applied successfully to the determination of FA in various types of matrices. In a first step, the exposure of SPME fiber to the headspace of a vial containing the derivatizing reagent allows the adsorption of the reagent. Subsequently, the FA present in the headspace of the food, biological or environmental sample is extracted by directly reacting with PFBHA on the fiber, which is then injected into the gas chromatograph.

This method uses no solvents, is completely reusable, extremely accurate, and reproducible, and requires no sample preparation steps. However, as several factors can impact the extraction efficiency of the FA, it is necessary to optimize the HS-SPME procedure. In this study, the optimization steps for the extraction and desorption conditions were performed by using the real matrix, in order to ensure the best extraction efficiency and linearity.

### Performance evaluation

In order to verify the performance of the proposed method under the optimized conditions, we studied the parameters necessary for this purpose: linear range, limit of detection (LOD), limit of quantification (LOQ), inter- and intra-day precision, and accuracy at different concentration levels.

The linearity of the proposed method was investigated over a concentration range of 0.2–1.20 µg mL^−1^, corresponding to the expected variation of FA content in the unknown urine samples [24–26]. An external calibration curve with six spiked calibration standards covering the aforementioned range was constructed by plotting the peak area versus the nominal concentration of each calibration solution. For each calibration level, 3 spiked solutions were prepared and analyzed in order to generate an average value for each point. Least-squares linear regression analysis was applied to get the best fitting function between the experimental data points. Thus, the calculated six-point linear plots were used for the quantification of analytes. LOD and LOQ were calculated as the smallest amount of FA-oxime given an S/N ≥ 3 and an S/N ≥ 10, respectively.

Method precision was evaluated as intra- and inter-day repeatability and expressed as coefficient of variation (CV%). Intra-day precision was assessed by analyzing five replicates at three concentration levels (0.25, 0.53, and 0.95 µg mL^−1^), while inter-day precision was determined from triplicate analyses of the same levels over three different days. Accuracy was evaluated as percent recovery (%Rec) using ten blank urine samples spiked at 0.53 µg mL^−1^.

### Workers’ urine collection, covariates and statistical analysis

To evaluate the applicability of the developed HS-SPME GC–MS/MS method under real occupational conditions and to explore its potential use for biological monitoring, urine samples were collected from workers occupationally exposed to FA and from non-exposed subjects, mainly administrative staff, who served as the reference group. The biomonitoring campaign was conducted during the 2024–2025 academic year in public hospital pathology departments and in a fire department located in central Italy.

Occupationally exposed workers included pathologists, laboratory technicians, and researchers employed in histopathology units, where FA in liquid form (formalin) is routinely used as a tissue fixative during diagnostic and research activities [27], as well as firefighters potentially exposed to FA as a component of combustion products generated during fire events [28, 29].

In hospital pathology and histopathology laboratories, occupational exposure to FA has been consistently documented during routine tasks such as specimen grossing, handling of formalin-fixed tissues, and histological processing. Air monitoring studies conducted in these settings have reported measurable airborne FA concentrations during grossing and specimen handling, with typical levels ranging from approximately 0.02 to 0.33 ppm in pathology departments and mean personal exposures around 0.16 ppm with short-term peak (ceiling) concentrations reaching up to approximately 1.1 ppm under routine working conditions [30, 31]. Firefighters may also be exposed to FA as a component of combustion products generated during fire events, as volatile organic compounds including FA have been detected at elevated concentrations in the atmosphere of fire training and operational environments. Personal air monitoring studies of firefighters have reported FA concentrations ranging from approximately 0.1 to 8.3 ppm in field exposures, with some measurements approaching or exceeding short-term exposure limits during active firefighting, and concentrations up to 9.8 mg/m^3^ during specific phases of fire suppression [32]. Additionally, assessments of wildland fire smoke have identified FA at levels from approximately 0.048 to 0.42 mg/m^3^ (0.04–0.35 ppm), with mean values around 0.16 mg/m^3^ (0.13 ppm) in combustion plumes, underscoring the presence of FA in fire smoke and the potential for occupational inhalation exposure in fireground conditions [33].

Against this background of documented but heterogeneous occupational exposure scenarios, characterized by variable intensity, intermittent peaks, and effective implementation of control measures in many settings, the need for reliable and sensitive biological monitoring tools remains a relevant issue. The present study was therefore conducted within the framework of the BRIC 2022 project (Research and Development for Large Projects - 2022) promoted by the Italian National Institute for Insurance against Accidents at Work (INAIL), in collaboration with research and academic institutions with the aim of developing and applying an analytical approach suitable for assessing internal FA exposure under real-world occupational conditions. Before starting, the study protocol, titled “Experiment aimed at preventing and managing the risk of FA exposure in healthcare and other workplace settings through the creation of a network of IRCCS and hospitals and/or other partners for the recruitment of exposed workers”, was approved by the Ethics Committee of the coordinating Oncology Unit and conducted in accordance with the Declaration of Helsinki and subsequent amendments. Written informed consent was obtained from all participants.

Before agreeing to participate, all workers received information about the aims and plans of the research other than the modalities to compile the questionnaire and to collect and store urine samples. On the sampling day, participants completed a structured questionnaire collecting information on sociodemographic characteristics and potential determinants of internal FA levels, including age, sex, smoking habits, body weight, and height. Then, at the end of the work shift, each participant provided a spot urine sample in a benzene-free polypropylene container and the fulfilled documents to the research team. Biological samples were then immediately stored into refrigerated containers, transported to the laboratory and immediately partitioned into glass vials, as described above. Then, the vials were coded and frozen at − 20 °C, until analysis (within 30 days). This stringent pre-analytical protocol is consistent with standard procedures adopted in biomonitoring investigations, in accordance with the standardized guidelines for diagnostic samples described by Guder et al. [33]. According to these clinical chemistry benchmarks, maintaining samples at −20 °C is the established practice to effectively prevent enzymatic activity and significantly slow down chemical degradation processes that could otherwise compromise the quantification of volatile and reactive analytes [34]. Although a formal longitudinal validation study would provide additional robustness, preliminary tests conducted in our laboratory confirmed that urinary FA levels remained stable for at least 3 months at investigated concentrations under the applied storage conditions.

Urinary FA concentrations were investigated in relation to occupational exposure status and selected covariates (sex, age, body mass index, and smoking habits) to explore potential determinants of internal FA levels and sources of inter-individual variability.

Descriptive statistics were calculated for all variables. Continuous variables were summarized using medians and interquartile ranges (IQR), while categorical variables were expressed as absolute numbers and relative percent frequencies. The distribution of urinary FA concentrations was assessed using the Shapiro–Wilk test and Q–Q plots, revealing a non-normal distribution. Accordingly, non-parametric statistical methods were applied. Comparisons between two independent groups were performed using the Mann–Whitney U test, while differences among more than two categories were evaluated using the Kruskal–Wallis test. To further explore associations between urinary FA concentrations and the investigated covariates, correlation analyses were performed using Spearman’s rank correlation coefficient. Missing data were handled using pairwise deletion and a two-tailed p-value < 0.05 was considered statistically significant. All statistical analyses were carried out using IBM SPSS Statistics software (version 25, IBM Corp., Armonk, NY, USA) 

## Results and discussion

### Implemented method

#### Sampling “on-fiber derivatization” procedure

The implemented analytical procedure was developed through a systematic investigation of the main experimental variables affecting the HS-SPME on-fiber derivatization process, with particular attention to temperature and extraction/derivatization time, with the specific objective of obtaining a robust and reproducible method suitable for biological monitoring applications. The optimization strategy was therefore oriented not only toward maximizing analytical sensitivity, but also toward ensuring method stability, repeatability, and operational simplicity, which are essential requirements for routine occupational health surveillance.

The choice of the SPME fiber coating was guided by its affinity toward the target analyte and the derivatization reagent, as well as by performance-related criteria relevant to biomonitoring, such as reproducible reagent uptake, consistent desorption behavior, and long-term analytical reliability. A critical evaluation of the scientific literature highlighted the suitability of polydimethylsiloxane/divinylbenzene (PDMS/DVB) fibers for PFBHA-based on-fiber derivatization, due to their balanced adsorbent properties and convenient chromatographic performance [12, 35–37]. On this basis, PDMS/DVB was selected as the optimal coating for urinary FA determination.

Carboxen-containing coatings, such as CAR/PDMS, although characterized by a high surface area and strong adsorption of low-molecular-weight compounds, were intentionally excluded. Several studies have reported limitations associated with their use in PFBHA on-fiber derivatization of FA, including the formation of undesired by-products during thermal desorption, increased peak tailing and memory effects, and reduced reproducibility of PFBHA–oxime desorption [36, 37]. These effects may compromise quantitative accuracy and precision, particularly at trace concentration levels, and are therefore incompatible with the analytical requirements of biological monitoring, where reliable comparison of results across time and individuals is crucial. The selection of PDMS/DVB was thus essential in achieving the satisfactory precision and recovery values reported in the validation study.

Regarding the experimental conditions, prior to the sampling-derivatization phase, all thawed urine samples (reagents, blanks, calibration standards, and biological samples) were equilibrated at 30 °C for at least 30 min to ensure a stable partitioning between the liquid and vapor phases. This step contributed to minimizing matrix-related variability, as reflected in the low intra- and inter-day CV% values obtained during method validation. After equilibration, the SPME fiber was exposed to the headspace of a vial containing approximately 5 mg of pure PFBHA for 20 s. This exposure time was optimized to ensure sufficient and reproducible reagent loading, while preventing fiber overloading, thereby improving repeatability and avoiding additional conditioning steps.

Subsequently, the reagent-impregnated fiber was exposed to the headspace of the urine sample for 20 min, allowing FA to react directly with PFBHA on the fiber surface. The selected reaction time ensured quantitative and reproducible derivatization and extraction of FA, as confirmed by the recovery and precision data obtained at all tested concentration levels. After sampling, the fiber was directly introduced into the GC injector for thermal desorption and GC–MS/MS analysis, contributing to the low limits of detection and quantification achieved by the method.

Overall, the combination of in situ derivatization, headspace extraction, and direct GC–MS/MS analysis provides a solvent-free and minimally invasive sample preparation strategy that limits sample manipulation and potential contamination. The simplicity of the workflow, together with the limited number of critical steps and the short analytical time, was specifically designed to meet the practical requirements of biological monitoring in occupational health surveillance.

#### GC–MS/MS detection

The GC–MS/MS conditions were optimized to ensure adequate chromatographic resolution, high selectivity, and stable analytical response, in line with the requirements of trace-level determination of FA in complex biological matrices. Method optimization was initially performed by repeated injections of PFBHA and FA-oxime obtained from the reaction of the pure derivatization reagent with a diluted formalin solution (1 µg mL^−1^), following the procedure described above.

Chromatographic separation and retention time optimization were first carried out in full scan mode (m/z 50–350, 1.6 scans s^−1^) using a working standard solution (6 µg mL^−1^). Under these conditions, analytes were unambiguously identified based on their retention times and mass spectral labelling. The finalized injector program involved a programmed temperature vaporization (PTV) starting at 60 °C (0.1 min), followed by heating to 220 °C at 5 °C s^−1^ (1 min hold). An additional automated fiber cleaning step was included at 250 °C for 15 min to prevent carryover effects and ensure analytical repeatability, which is particularly relevant for routine biological monitoring applications.

The oven temperature program was set from 60 °C (4 min hold) to 90 °C at 15 °C min^−1^ (4 min hold), then increased to 160 °C at 10 °C min^−1^, and finally to 250 °C at 25 °C min^−1^ (2 min hold). The transfer line temperature was maintained at 280 °C, and helium was used as carrier gas at a constant flow rate of 1.1 mL min^−1^. Under these conditions, the total chromatographic run time was 22 min, representing a suitable compromise between chromatographic performance and analytical throughput.

Quantification of FA-oxime was performed in tandem mass spectrometry mode using multiple reaction monitoring (MRM). Collision energies for each transition were optimized in the range of 5–50 eV through repeated injections of both the derivatization reagent and the FA-oxime derivative. The most abundant fragment ion was selected as precursor ion, and a collision energy of 15 eV was identified as optimal for generating stable and intense product ions. Dwell times were set to 10 ms to ensure sufficient sensitivity while maintaining fast data acquisition. Among the characteristic product ions, the most intense transition was selected for quantification, while a second transition was used for qualitative confirmation.

**Fig. 1:**
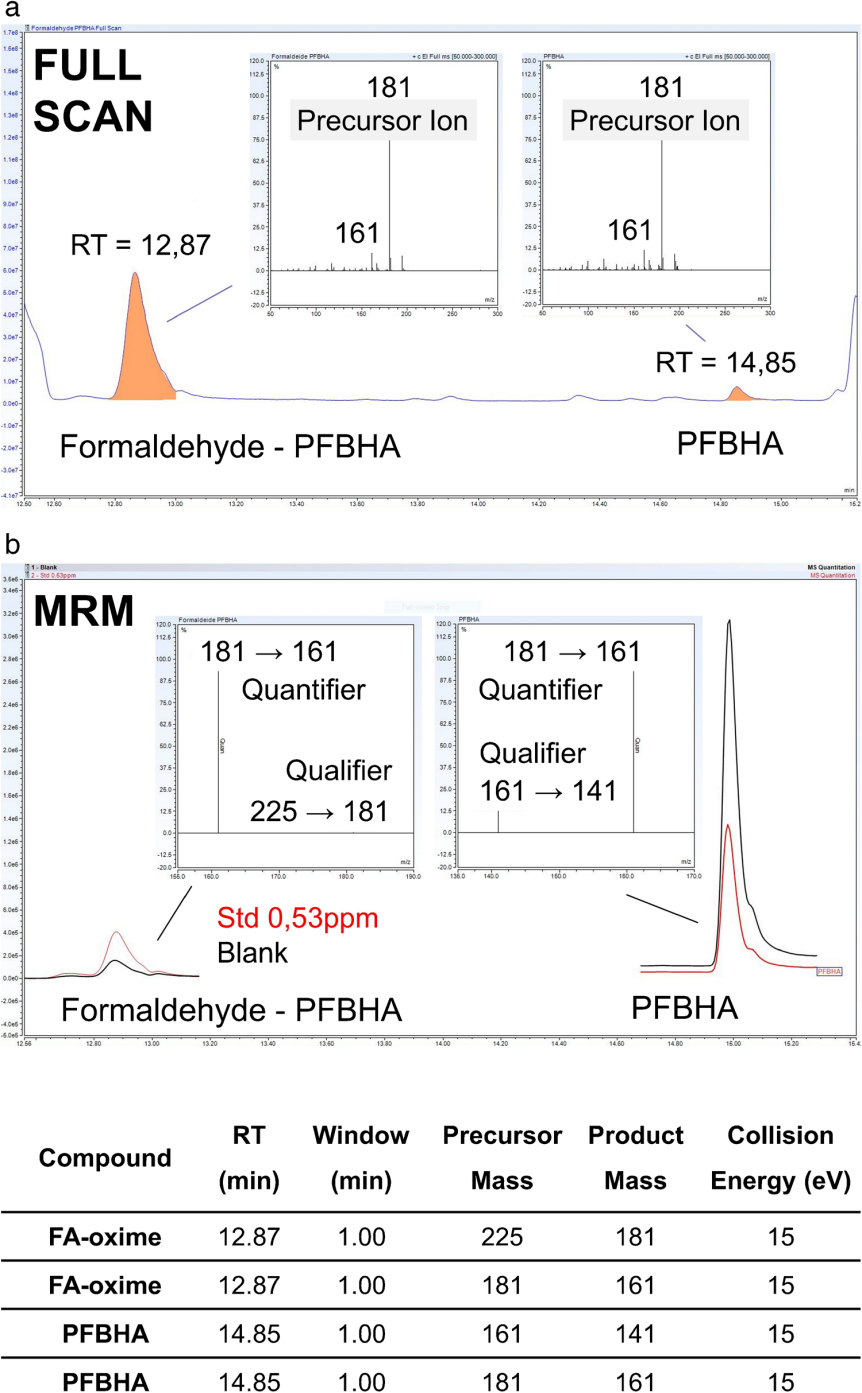
Chromatograms of urine blank and calibration standard solution acquired under working conditions. **a** GC–MS (full scan) chromatogram of working standard solution (6 µg mL^−1^) and its relative mass spectra for PFBHA and FA-oxime; **b** GC–MS/MS (MRM) overlayed chromatograms of working standard solution (0.53 µg mL^−1^) and relative transitions mass spectra with optimized instrumental parameters

After optimization, the mass spectrometer was operated in time-scheduled MRM mode, acquiring data within a defined time window assigned to the retention time of each compound. A cycle time of 10 cycles s^−1^ and a time window of ±0.5 min around each analyte retention time were applied, allowing efficient data acquisition while minimizing background noise and matrix interferences. Representative chromatograms acquired under the optimized conditions together with the MRM transitions for GC–MS/MS are shown in Fig. 1.

Figure 1a reports the GC–MS full scan chromatogram of a working standard solution (6 µg mL^−1^) together with the corresponding mass spectra of PFBHA and FA-oxime. Figure 1b shows the overlaid GC–MS/MS MRM chromatograms of a calibration standard (0.53 µg mL^−1^) and a urine blank, highlighting the selectivity achieved by tandem mass spectrometric detection. The molecular ions identified in full scan mode were subsequently used as precursor ions for MRM acquisition, and the optimized transitions enabled selective detection of FA-oxime even in the presence of excess derivatization reagent and endogenous urinary components.

The high specificity provided by triple quadrupole detection in MRM mode is particularly advantageous for the analysis of complex biological matrices such as urine. By effectively reducing matrix-related interferences, this approach represents a fundamental prerequisite for generating reliable quantitative data in biological monitoring studies, especially when low analyte concentrations and high sample variability are expected.

### Method validation

#### Analytical performance

After optimization of the HS-SPME sampling procedure and GC–MS/MS detection conditions, the analytical performance of the method was systematically evaluated in the urine matrix to assess its sensitivity, precision, and quantitative reliability.

The main validation parameters, including determination coefficient (R^2^), limit of detection (LOD), limit of quantification (LOQ), coefficient of variation (CV%), and recovery (%Rec), are summarized in Table 1.

**Table 1:** Analytical performance parameters provided from the present method: average recovery (Rec%) and coefficient of variation (CV%) at three concentration levels (0.25–0.53–0.95 µg mL^−1^), determination coefficients (R^2^), and limits of detection (LOD) and quantification (LOQ) for the determination of urinary FA using the proposed method

Compound	Range (mg/L)	Day 1		Day 2		Day 3		Inter-day		*R*^2^	LOD (mg/L)	LOQ (mg/L)
		CV%	Rec%	CV%	Rec%	CV%	Rec%	CV%	Rec%			
FA-oxime	0.25	5.55	89.27	5.40	114.46	3.85	86.26	6.94	101.25	0.99	0.03	0.11
	0.53	0.93	89.26	5.09	124.20	4.04	81.16	4.04	81.16			
	0.95	2.66	96.90	2.16	88.63	2.59	102.77	2.30	88.50			

The analytical validation of the HS-SPME GC–MS/MS method confirmed its strong quantitative performance in a complex biological matrix.

Direct on-fiber derivatization combined with MS/MS detection provided high sensitivity, characterized by low LOD and LOQ values (0.03 and 0.11 µg mL^−1^). Calibration curve [Y = (19787 ± 528) X + (13046 ± 368)] showed excellent goodness of fit, with an R^2^ of 0.99 across the investigated concentration range. For an operational perspective, the achieved sensitivity is particularly relevant in view of the stringent occupational exposure limits currently established for FA, which require analytical methods adequate for detecting very low internal dose biomarkers. In this respect, the proposed procedure meets this requirement, enabling the quantification of urinary FA at trace concentration levels compatible with occupational exposure assessment.

Method precision was satisfactory across all tested concentration levels (0.25, 0.53, and 0.95 µg mL^−1^), with intra-day and inter-day CV% values generally below 7%, indicating good repeatability and intermediate precision. Recovery values showed some variability across concentration levels and analytical days, ranging overall from approximately 81% to 124%, which is acceptable for trace-level determinations in complex biological matrices and reflects the inherent variability associated with headspace extraction and on-fiber derivatization processes.

Overall, the validated performance parameters confirm that the proposed HS-SPME GC–MS/MS method represents a reliable and sensitive analytical tool for urinary FA determination, suitable for application in biological monitoring studies and occupational exposure investigations.

#### In field application—urinary FA levels

To explore the potential of the proposed method for biological monitoring purposes, the developed HS-SPME GC–MS/MS method was applied to the determination of urinary FA in a population of workers recruited from laboratory, clinical, and office workstations of the participating hospitals, as well as from a fire station, within the framework of an occupational biomonitoring campaign. The main sociodemographic and lifestyle characteristics of the study population are summarized in Table 2.

A total of 124 subjects participated in the study, including 82 workers occupationally exposed to FA and 42 administrative employees not occupationally exposed who served as the reference group.

Overall, the study population had a median age of 49 years (IQR 19), with exposed workers being generally older than controls (median 50 vs 38.5 years, respectively). Males were slightly more represented among exposed workers (56.1%), whereas females were more prevalent in the control group (52.4%).

Regarding body mass index (BMI), most participants who provided anthropometric information fell within the normal weight category, although a substantial proportion of missing BMI data was observed in both groups.

Smoking habits were broadly comparable between exposed and non-exposed subjects. Non-smokers represented the majority of participants in both groups (approximately 61%), while current smokers accounted for 28.0% of exposed workers and 23.8% of controls.

**Table 2:** Sociodemographic and lifestyle characteristics of the study population

Variable	Exposed workers (*n* = 82)	Controls (*n* = 42)	Total (*n* = 124)
Age, years			
^1^Median (IQR)	50 (15)	38.5 (23)	49 (19)
Sex, *n* (%)			
Male	46 (56.1)	20 (47.6)	66 (54.8)
Female	36 (43.9)	22 (52.4)	58 (45.2)
^2^Body mass index, *n* (%)			
Underweight (≤ 18.49)	2 (2.4)	2 (4.8)	4 (3.2)
Normal weight (18.5–24.99)	22 (26.8)	13 (31.0)	35 (28.3)
Overweight (25–29.99)	13 (16.0)	3 (7.1)	16 (12.9)
Obesity (≥ 30)	4 (4.8)	0 (0)	4 (3.2)
Unknown	41 (50.0)	24 (57.1)	65 (52.4)
Smoking habit, *n* (%)			
Non-smokers	50 (61.0)	26 (61.9)	76 (61.3)
Current smokers	23 (28.0)	10 (23.8)	33 (26.6)
Ex-smokers	6 (7.3)	3 (7.1)	9 (7.3)
Unknown	3 (3.7)	3 (7.1)	6 (4.8)

^1^Age is reported as median and interquartile range (IQR); ^2^*BMI*, body mass index (weight in kg/height squared in m)

Based on these characteristics, urinary FA concentrations were evaluated to assess potential differences between occupationally exposed workers and controls and to explore potential determining factors of internal FA levels. Table 3 reports the urinary FA concentrations of investigated variables, expressed as median and interquartile range due to their non-normal distribution, together with the non-parametric statistical tests applied. Urinary FA concentrations showed a wide inter-individual variability in both occupationally exposed workers and controls. No statistically significant difference in urinary FA concentrations was observed between exposed and non-exposed subjects (median 0.20 vs 0.17 µg mL^−1^, respectively; p = 0.48).

Similarly, no significant associations were found with age class (p = 0.91), BMI category (p = 0.38), or smoking status (median 0.23 µg mL^−1^ in smokers vs 0.19 µg mL^−1^ in non-smokers; p = 0.55). Although a trend toward higher median FA levels was observed in subjects with BMI ≥ 25 kg m^−2^, the limited number of cases in the underweight category and the overall variability did not allow for statistically significant differences to be detected.

A statistically significant difference was observed according to sex, with males showing higher urinary FA concentrations compared to females (0.24 vs 0.15 µg mL^−1^, p = 0.0045).

In agreement with our findings, Chrostek et al. [37] in previous biomonitoring studies reported sex-related variability in aldehyde metabolism and excretion, potentially related to differences in enzymatic activity, body composition, and hormonal regulation. In particular, gender-related differences in hepatic activities of alcohol and aldehyde dehydrogenases have been reported in humans, with certain ADH isoenzymes showing higher activity in males than females, potentially affecting the biotransformation of endogenous aldehydes such as FA [38]. Additionally, population-based data suggest that serum aldehyde levels exhibit sex-specific associations with sex steroid hormones [39]. Although these mechanisms were not specifically addressed in the present study, they highlight biologically plausible pathways for sex-dependent variability in urinary aldehyde biomarkers.

In addition to sex-related biological variability, lifestyle-related factors were explored as potential contributors to inter-individual differences in urinary FA concentrations. Smoking status emerged as a relevant determinant of urinary FA levels. Among smokers, urinary FA concentrations tended to be higher compared to non-smokers, and differences between exposed and non-exposed subjects were more evident within this subgroup, although variability remained high. In fact, when the analysis was restricted to smokers only, occupationally exposed subjects showed higher median urinary FA concentrations compared to smoking controls (0.24 vs 0.19 µg mL^−1^, respectively). However, this difference did not reach statistical significance (Mann–Whitney U test, p = 0.12), likely due to the limited sample size of the smoking control group.

Conversely, among non-smokers, urinary FA concentrations were comparable between exposed workers and controls, suggesting that lifestyle-related sources may contribute substantially to the internal FA burden and warrant further investigation in larger study populations.

**Table 3:** Urinary FA concentrations [µg mL^−1^] according to some characteristics of participants

Variable		[FA] µg mL^−1^ M^1^ (IQR)^2^	*p*
	Whole studied population	0.20 (0.18)	
	Participants exposed to FA	0.20 (0.15)	0.48^3^
	Participants not exposed to FA	0.17 (0.10)	
Age	≤ 40 > 40	0.21 (0.19) 0.19 (0.16)	0.91^3^
Gender	Male Female	0.24 (0.10) 0.15 (0.13)	**0.0045**^**3**^ **(ρ = −0.279, p = 0.004)**^**4**^
BMI^5^	Underweight (BMI ≤ 18.49) Normal weight (18.5 ≤ BMI < 25) Over/obese (BMI ≥ 25)	0.13 (0.09) 0.15 (0.10) 0.20 (0.10)	0.38^6^
Smoking habits	Non-smokers Smokers	0.19 (0.17) 0.23 (0.18)	0.55^3^
Smoker	Exposed to FA Not exposed to FA	0.24 (0.14) 0.19 (0.12)	0.12^3^

^1^*M*, median; ^2^*IQR*, interquartile range; ^3^Mann–Whitney U test; ^4^Spearman’s rank correlation coefficients; ^5^*BMI*, body mass index (weight in kg/height squared in m); ^6^Kruskal–Wallis test

A correlation analysis was performed to further explore the relationships between urinary FA concentrations and the investigated covariates.

Spearman’s rank correlation coefficients confirmed the absence of significant correlations between urinary FA levels and occupational exposure status, age, BMI, or smoking habits (p > 0.05). In contrast, a weak but statistically significant negative correlation was observed between urinary FA concentrations and sex (ρ = −0.279, p = 0.004), indicating higher FA levels in male subjects. This finding is consistent with the results of the group comparisons and supports the role of sex-related factors in contributing to inter-individual variability of urinary FA levels.

Although quantitative data on urinary biomarkers of FA exposure in occupational settings remain relatively limited, available studies consistently report substantial overlap between exposed and non-exposed populations, particularly at low exposure levels and under controlled working conditions. In this context, the absence of statistically significant differences in urinary FA concentrations between occupationally exposed workers and controls observed in the present study is consistent with previous biomonitoring investigations.

For instance, Motta et al., in a combined environmental and biological monitoring study conducted among healthcare workers in an anatomical pathology unit, reported generally low urinary FA concentrations (median range: 0.22–0.56 µg mL^−1^), despite measurable airborne exposure. Environmental monitoring showed that FA concentrations were typically low, although localized measurements slightly exceeded the NIOSH recommended exposure limit of 0.1 ppm, with peak values of approximately 0.16 ppm recorded in the grossing room during specimen handling. Although the study did not include an external non-exposed control group, repeated measurements revealed a modest weekly accumulation, underscoring the difficulty of discriminating low-level occupational exposure using urinary FA biomarkers alone [40].

More broadly, several authors have highlighted the intrinsic limitations of urinary FA as a biomarker of exposure, particularly in the absence of high or acute occupational exposure scenarios. This limited discriminative power is largely attributable to the ubiquitous nature of FA and its substantial endogenous background. Urinary FA concentrations reflect the combined contribution of endogenous metabolic production, environmental exposure, lifestyle-related factors, and occupational sources, resulting in considerable inter-individual variability that may obscure modest exposure-related differences in biomonitoring studies [41, 42]. Consistently, recent systematic reviews addressing occupational FA exposure have shown that increased urinary biomarker levels are reported in only about half of the evaluated studies, with outcomes strongly influenced by individual susceptibility and external factors beyond workplace exposure, particularly at low exposure levels [8, 43].

In parallel, several alternative biomarkers have been proposed to investigate FA exposure and its biological interactions, including DNA adducts, hemoglobin adducts, and specific urinary metabolites formed through reactions with nucleophilic cellular components. For example, Wang et al. [43] reported markedly higher levels of a formaldehyde–DNA adduct in leukocytes of smokers compared to non-smokers [44]. These biomarkers reflect different biological processes compared to urinary FA. DNA and protein adducts represent molecular interactions with cellular macromolecules (biomarkers of effects) and may therefore provide information on biological reactivity rather than on the direct systemic levels of the parent compound. Moreover, toxicokinetic studies have shown that formaldehyde–DNA adducts can also originate from endogenous FA generated during normal cellular metabolism, which may complicate their interpretation in exposure assessment studies [45]. In addition to the parent compound, sulfur-containing metabolites formed through reactions of FA with glutathione or other thiol-containing compounds (e.g., cysteine or cysteinylglycine) have been proposed as potential exposure-related biomarkers, including thioproline and thioprolinyl-glycine, which can be detected and quantified in human urine [46–48]. Urinary formate, a downstream oxidation product of FA metabolism, proposed in some studies, lacks specificity for FA exposure, as it can originate from multiple endogenous metabolic pathways and from other environmental sources [49]. In this perspective, different biomarkers—including urinary FA, specific metabolites, and adduct-based markers—should be considered complementary tools for investigating different aspects of FA exposure and biological response.

Overall, these findings highlight the complexity of interpreting urinary FA as a biomarker of occupational exposure, particularly under low and well-controlled exposure conditions. Given the substantial endogenous background of FA, its rapid metabolic turnover and the relatively low airborne concentrations typically encountered in modern occupational environments, the contribution of low-level external exposure may be difficult to distinguish from physiological variability in population-based biomonitoring studies, resulting in a considerable overlap between exposed and non-exposed subjects. In this context, the absence of clear exposure-related differences should not necessarily be interpreted as a lack of relevance of the biomarker, but rather as a reflection of the biological characteristics of this endogenous compound and the limitations inherent to its use at low exposure levels.

At the same time, the proposed HS-SPME GC–MS/MS method demonstrated the capability to reliably quantify very low urinary FA concentrations in real-world biological samples across a broad concentration range. This supports its applicability in biological monitoring studies aimed at characterizing internal exposure and determinants of inter-individual variability, as well as for investigating occupational scenarios characterized by higher or more variable exposure levels, where biomarker-based assessments may provide more informative exposure indicators.

## Conclusions

The present study has developed and validated a specific analytical method for the determination of pure FA in the urine of professionally exposed workers, addressing a topic of great importance for workplace health protection. FA, a substance classified as a carcinogen and widely used in various production sectors, requires careful monitoring that integrates information from environmental and personal monitoring with objective data on actual absorption by the body. The limited discriminatory power of urinary FA as an exposure biomarker is largely attributed to its short half-life, contributions from endogenous metabolism, and interference from personal and environmental factors, making it a challenging measure for low-level occupational exposure compared to direct air monitoring or other biomarkers like protein adducts.

In this context, developing a reliable analytical protocol represents an important step toward a more comprehensive and scientifically sound risk assessment. The entire development process—from defining optimal sampling and storage conditions, to selecting the most effective reagents and derivatizations, to instrumental calibration and validation—led to the development of a method with performance characteristics suited to the needs of health surveillance. The validation parameters demonstrated good linearity within the concentration range of interest, satisfactory intra- and inter-day precision, and detection and quantification limits low enough to allow the identification of trace amounts of the substance even under moderate exposure conditions.

Among the method’s main strengths is its high sensitivity, which allows for the quantification of FA as such at very low levels, thus ensuring accurate assessment even for workers exposed to low but still toxicologically relevant concentrations. Furthermore, the excellent selectivity achieved by optimizing the analysis conditions reduces the risk of interference from urinary tracts and allows for a clean reading of the analytical signal. The method’s reproducibility is also a strong point, as it allows for reliable results over time and under different operating conditions, making the protocol applicable not only in experimental studies but also in routine health surveillance programs.

However, the method is not without limitations. The high reactivity and volatility of FA require rigorous sample management, with collection, storage, and transport conditions strictly controlled to prevent degradation or loss of the substance. Furthermore, the concentration of FA in urine can be influenced by individual biological variables, such as metabolism, hydration, and elimination time. These factors can introduce variability in results and require integration with anamnestic data or the determination of complementary biomarkers. The need for advanced analytical instrumentation and highly qualified personnel also represents a potential limitation, as it could limit the method's uptake in laboratories with limited resources or inexperienced personnel. Despite these critical issues, the developed method proves to be a valid, technically robust, and scientifically sound tool for assessing FA exposure in occupational settings. Its practical applicability and ability to provide direct information on FA absorption make it an important tool for health surveillance and the prevention of occupational risks. Looking ahead, further research could focus on extending the method to samples from larger and more diverse populations, standardizing sampling procedures, and exploring the potential for integrating it with new biomarkers of effect or exposure. A multidimensional and integrated approach could therefore contribute to improving the quality of risk assessments, providing a completer and more reliable picture to protect the health of exposed workers.

## Data Availability

The data that support the findings of this study are not openly available due to reasons of sensitivity and are available from the corresponding author upon reasonable request.
